# Observation of a high degree of stopping for laser-accelerated intense proton beams in dense ionized matter

**DOI:** 10.1038/s41467-020-18986-5

**Published:** 2020-10-14

**Authors:** Jieru Ren, Zhigang Deng, Wei Qi, Benzheng Chen, Bubo Ma, Xing Wang, Shuai Yin, Jianhua Feng, Wei Liu, Zhongfeng Xu, Dieter H. H. Hoffmann, Shaoyi Wang, Quanping Fan, Bo Cui, Shukai He, Zhurong Cao, Zongqing Zhao, Leifeng Cao, Yuqiu Gu, Shaoping Zhu, Rui Cheng, Xianming Zhou, Guoqing Xiao, Hongwei Zhao, Yihang Zhang, Zhe Zhang, Yutong Li, Dong Wu, Weimin Zhou, Yongtao Zhao

**Affiliations:** 1 MOE Key Laboratory for Nonequilibrium Synthesis and Modulation of Condensed Matter, School of Physics, Xi’an Jiaotong University, Xi’an, 710049 China; 2 Science and Technology on Plasma Physics Laboratory, Laser Fusion Research Center, China Academy of Engineering Physics, Mianyang, 621900 China; 3 Institute for Fusion Theory and Simulation, Department of Physics, Zhejiang University, Hangzhou, 310058 China; 4 Xi’an Technological University, Xi’an, 710021 China; 5 Institute of Applied Physics and Computational Mathematics, Beijing, 100094 China; 6 Graduate School, China Academy of Engineering Physics, Beijing, 100088 China; 7 Institute of Modern Physics, Chinese Academy of Sciences, Lanzhou, 710049 China; 8 Xianyang Normal University, Xianyang, 712000 China; 9 Beijing National Laboratory for Condensed Matter Physics, Institute of Physics, Chinese Academy of Sciences, Beijing, 100190 China; 10 School of Physical Sciences, University of Chinese Academy of Sciences, Beijing, 100049 China

**Keywords:** Nuclear fusion and fission, High-field lasers, Laser-produced plasmas

## Abstract

Intense particle beams generated from the interaction of ultrahigh intensity lasers with sample foils provide options in radiography, high-yield neutron sources, high-energy-density-matter generation, and ion fast ignition. An accurate understanding of beam transportation behavior in dense matter is crucial for all these applications. Here we report the experimental evidence on one order of magnitude enhancement of intense laser-accelerated proton beam stopping in dense ionized matter, in comparison with the current-widely used models describing individual ion stopping in matter. Supported by particle-in-cell (PIC) simulations, we attribute the enhancement to the strong decelerating electric field approaching 1 GV/m that can be created by the beam-driven return current. This collective effect plays the dominant role in the stopping of laser-accelerated intense proton beams in dense ionized matter. This finding is essential for the optimum design of ion driven fast ignition and inertial confinement fusion.

## Abstract (web summary)

A detailed understanding of particle stopping in matter is essential for nuclear fusion and high energy density science. Here, the authors report one order of magnitude enhancement of intense laser-accelerated proton beam stopping in dense ionized matter in comparison with currently used models describing ion stopping in matter.

## Introduction

Alpha-particle stopping in dense ionized matter is essential to achieve ignition in inertial confinement fusion^1–5^. Fast ignition (FI) relies even more on a detailed understanding of ultrahigh-current ion stopping in matter, which is therefore considered as a fundamental process of utmost importance to nuclear fusion. In the fast ignition scheme^6–9^, a short and intense pulse of energetic charged particles—electrons, protons, or heavy ions—generated by an ultra-high-intensity laser, is directed toward the pre-compressed fusion pellet. The charged-particle beam requirements to achieve ignition have been discussed and studied in detail previously^10–14^ based on single-particle stopping theory. However, the collective effects induced by high-current charged-particle beams could alter significantly the projected range, the magnitude of energy deposition, and therefore change the requirements for ignition correspondingly. Besides, in the cases of ion beam-driven inertial confinement fusion and high-energy density science, which require ultrahigh beam intensity from accelerators^15–19^, no collective effects on ion stopping processes due to high beam intensity are considered nor—to the best of our knowledge—were they reported in any previous experiments.

Since the discovery of alpha decay and the availability of energetic fission fragments, it became interesting to study fast particle stopping processes in matter^20–25^. In past decades, numerous theoretical models^26–32^, some of which can be considered to be further developments of the early work of Bethe^28^ and Bloch^29^, are built to describe individual charged particle stopping in dense ionized matter. Only recently experiments with sufficient precision were carried out with dense ionzied matter to distinguish between different models^25,33–35^. In these experiments, incident particles are generated from laser-induced nuclear reactions^33,34^, or from traditional accelerators^25,35^. Hence the beam intensity was low, and the individual ion stopping theories can be discriminated^36^.

Ultra-high-intensity lasers (10^18^–10^22^ W/cm^2^) have opened up perspectives in many fields of research and application^37–44^. By irradiating a thin foil with ultra-high-intensity lasers, an ultrahigh accelerating field (1 TV/m) can be formed and multi-MeV ions with high intensity (10^10^ A/cm^2^) in short timescale (~ps) are produced^45–53^. Such beams provide experimental opportunities to investigate the beam-driven complex collective phenomena^54–59^. In particular, the stopping power for these intense beam could be orders of magnitude higher than that for individual particles if the beam intensity is high enough^60–63^. In our previous experiment, we sent the laser-accelerated ion beams directly into the plasma target, and observed that the energy spectra of the ions were significantly downshifted after passing though the dense plasma^64^. This energy downshift was far beyond the Bethe–Bloch predictions (see Supplementary Figs. 8 and 9 for details). However, the large energy spread of the incident beam makes it difficult to correctly interpret the results.

In this article, we improve the precision of the measurement by using a magnetic dipole to trim out a quasi-monoenergetic proton beam. The dense ionized target is produced by irradiating a tri-cellulose acetate (TCA) foam sample with soft X-rays from a laser-heated hohlraum. Thus the temperature and density are homogeneous across the ionized target. This state can be maintained for a period of more than 10 ns. This period is two to three orders of magnitude longer than the beam duration and the beam-plasma interaction timescale. Therefore, the target can be considered to be quasi-static. This kind of experimental scenario allows for precise measurement of intense proton beam stopping in dense ionized matter. We observed that the energy loss is enhanced by one order of magnitude in comparison  to the predictions from individual-proton stopping theories, Bethe–Bloch^28,29^, Li–Petrasso (LP)^26^, standard stopping model (SSM)^32^. Through PIC simulation, we attribute the high degree of enhancement to a strong decelerating electric field induced by the intense proton beam. This collective effect is the primary cause for the enhanced stopping, and it is likely to have a major impact on nuclear fusion scenarios like fast ignition, alpha-particle self-heating, as well as ion driven inertial confinement fusion.

## Results

**Fig. 1:**
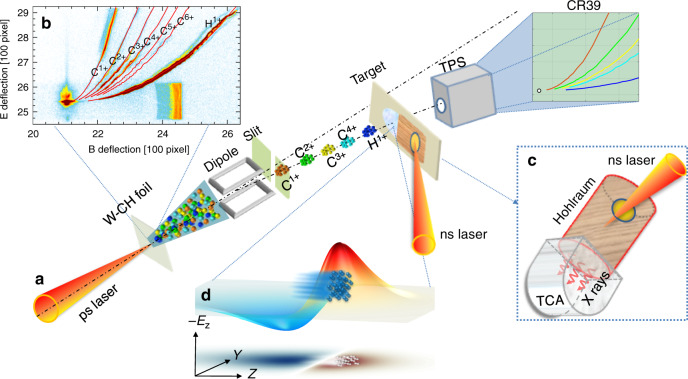
Layout of the experiment. **a** A ps laser is focused onto a tungsten foil, generating intense short-pulse ion beams with different species. A magnetic dipole with slits at the entrance and exit serve as **p**/*q* analyser to select monoenergetic ion beams. Such ions interact with the laser-generated plasma target and emerge from the target with a lower energy due to the incurred energy loss. The final-state energy is measured by a Thomson parabola in conjunction with CR39 film. **b** Parabola spectra of laser-accelerated ions without dipole measured by Thomson parabola in conjunction with Fuji image plate. **c** The target consists of a gold hohlraum converter to produce the soft X-rays that irradiate the TCA foam to generate a dense ionized sample. **d** The insert shows the simulation result of an intense proton beam moving along the *z* direction, inducing a strong longitudinal electric field, which is counter-directional to the proton beam propagation, causing the unusual high degree of stopping.

The experiment was carried out at the XG-III laser facility of Laser Fusion Research Center in Mianyang. The experimental layout is displayed in Fig. 1. Here a short and intense laser beam of 800 fs duration, 20 μm focal spot, and 150 J total energy irradiates a CH-coated tungsten foil (15-μm thick) to generate a charged-particle beam. The beam consists of a mixture of protons (H^1+^) and carbon ions with different charge states (C^1+^, C^2+^, C^3+^, and C^4+^). They originate at the backside of the target by means of the target normal sheath acceleration (TNSA). The predominant particle species is H^1+^, because the charge- to-mass ratio is maximum for this species, and is, therefore, more effectively accelerated than the lower charge-to-mass ratio species of carbon ions. The TNSA mechanism results in a broad range of particle energies, which is not favorable for quantitative analysis of the particle energy loss. A magnetic dipole, with entrance and exit slits, was used to generate a monoenergetic beam. The ions, spatially collimated by the 500 μm entrance slit, are dispersed laterally by the magnetic dipole according to their specific **p**/*q* value, where **p** and *q* are the particle momentum and charge, respectively. A second 500 μm exit slit, selects the quasi-monoenergetic ion pulses. The selected ions consist of different particle species, with similar **p**/*q* value, they have, however, different velocities and therefore arrive at the target pulse by pulse with different time delay. In the current case, the C^4+^ ion pulse lags behind proton pulse by about 30 ns at the plasma target, and C^3+^, C^2+^, and C^1+^ pulses are delayed more, therefore the laser-accelerated carbon ions have no influence on the proton beam stopping measurement.

A gold hohlraum converter was used to generate soft X-rays by interaction of a ns laser pulse (150 J), with the hohlraum walls. The X-rays subsequently irradiated and heated the foam target (C_9_H_16_O_8_, density of 2 mg/cm^3^ and thickness of 1 mm) to produce ionized matter. The hydrodynamic response of the heated foam target under this kind of scheme has been very well-investigated and the state has been well characterized^65–67^. Once heated by soft X-rays from hohlraum radiation, the material expansion occurs inside the target between the sponge-like structures. This micro expansion leads to target homogenization, while the volume and density of the entire target stay constant for more than 10 ns. Therefore, homogeneous, ns-long-living, and quasi-static ionized matter is generated. Unlike producing ionized matter through direct heating by high-power lasers—in which case strong electromagnetic fields are generated in the target and will greatly influence the proton transportation behavior^68–70^—here the electromagnetic fields resulted from the heating process can be neglected.

In order to determine plasma parameters, the emission spectra of the gold hohlraum and target matter were measured. The gold hohlraum radiation spectrum is well-represented by a 20 eV black body radiation spectrum, while the temperature of the plasma target is 17 eV. This value was obtained from a Boltzmann slope analysis of the He-like carbon lines. Given a temperature of 17 eV, and mass density of 2 mg/cm^3^, the number density of free electrons is determined to be 4 × 10^20^ cm^−3^ based on the FLYCHK code^71^.

**Fig. 2:**
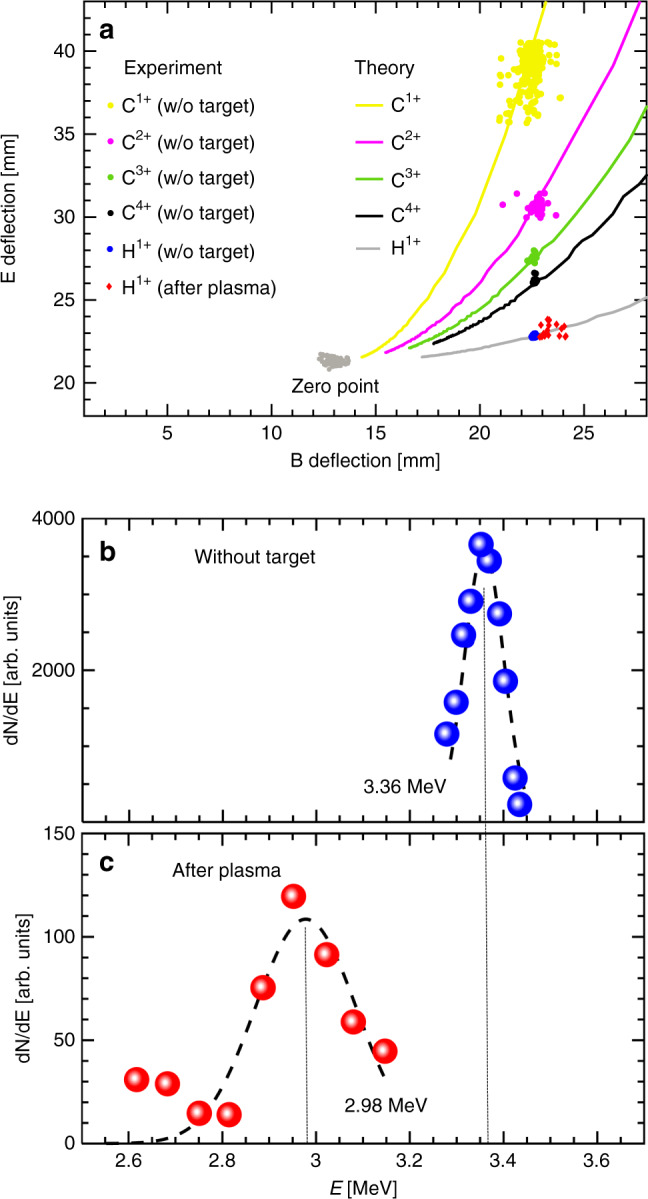
TPS CR39 tracks of quasi-monoenergetic ions passing through the system with/without target and the converted energies of protons. **a** Tracks of ions recorded on TPS CR39 passing through null target and plasma target. The *X* and *Y* coordinates represent the magnetic and electric deflection distances. The dots with the same B deflection distance from up to down (yellow, magenta, green, black, and blue in order) represent tracks formed by C^1+^, C^2+^, C^3+^, C^4+^, and protons passing through the system without (w/o) target. Tracks for protons passing through the plasma are represented by red dots. The tracks for the zero reference point are indicated with gray dots. The solid curves represent the theoretical tracks of these ion species with various energies. **b** Energy spectra of the protons passing the system without target. The experimental distribution (blue dots) is well fitted by a Gaussian profile (dashed line) with central energy of 3.36 MeV (dotted line). **c** Energy spectra of the protons passing the system without target. The experimental distribution (blue dots) is fitted by a Gaussian profile (dashed line) with central energy of 2.98 MeV.

A Thomson parabola spectrometer (TPS)^72–74^ in conjunction with a plastic track detector CR39 was used to obtain the energy spectrum of the charged particles. The energy resolution of the TPS achieves *E*/*δE* ~ 34 for protons at energy *E* = 3.36 MeV (see section I of the Supplementary information for details), where *δE* is the energy range covered by the incident beam spot on the detector. In Fig. 2a, tracks recorded on CR39 film are displayed for ions passing through the system with/without target. When the plasma target is inserted, only protons are observed in the TPS. The deflection distances of protons without/with target are converted to energies in Fig. 2b and c, respectively. The energy distribution of the incident, unperturbed protons without target centers at 3.36 MeV, and the full width at half maximum (FWHM) is about 0.10 MeV. After passing through the plasma target, the central energy is downshifted to 2.98 MeV and the FWHM increased to about 0.25 MeV.

## Discussion

The energy loss of intense ion beam in ionized matter is composed of two terms as d*E*/d*x* = (d*E*/d*x*)_collision _+ (d*E*/d*x*)_collective_. The first term (d*E*/d*x*)_collision_ describes the collisional stopping induced by binary interaction of the individual projectiles with the individual particles in the plasma. The second term (d*E*/d*x*)_collective_ describes the collective stopping induced by the beam-driven electric fields. (d*E*/d*x*)_collision_ consists of contributions from free electrons and plasma ions. The ionic contributions for partially ionized plasma include two parts, bound electrons and nuclei. In the current regime, where the protons are much faster than the thermal electrons, the contribution of the nuclei to (d*E*/d*x*)_collision_ can be neglected^75–77^. Here in this article the nuclear stopping is excluded.

**Fig. 3:**
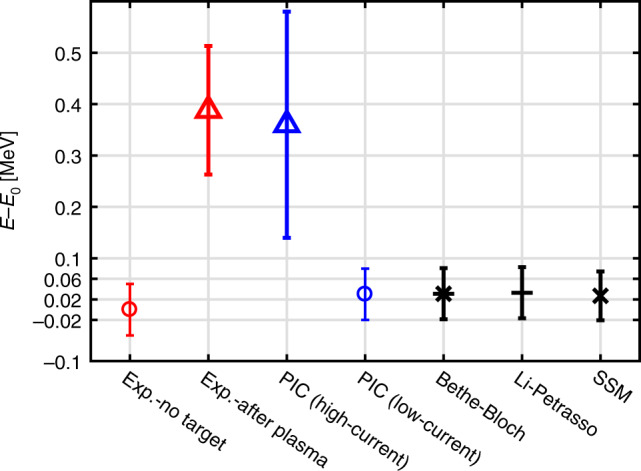
Measured (exp.-after plasma), numerical (PIC), and analytical predictions (Bethe–Bloch, SSM, and Li–Petrasso) of the proton beam energy spectra downshift after passing through the dense plasma target in relative to the central energy *E*_0_ of incident proton beam. The symbols represent the central energies and the bars represent the FWHMs of the respective spectra. For comparison, the data for the incident proton beam (no target) are shown as well.

In Fig. 3, the measured energy loss is compared to theoretical models, e.g., Bethe–Bloch model, Li–Petrasso (LP) theory, and SSM by Deutsch. These theories are based on binary collisions with free electrons, bound electrons, and/or plasmons. They all underestimate the measured stopping power by as much as one order of magnitude, even when considering an error of about 15% from the uncertainty of plasma electron density. We attribute this unusual high degree of stopping to collective electromagnetic effects induced by high-current ion beams.

In order to understand this enhanced stopping, both collective electromagnetic effects and close particle–particle interactions need to be taken into account. The most appropriate tool to simulate the conditions of the experiment is the particle-in-cell method (PIC), which in recent years has established itself as a state-of-the-art method for solving problems of kinetic plasma physics^54,78,79^.

We used the recently developed PIC code LAPINS^79,80^, which is able to simulate intense beam-plasma interaction in a self-consistent way, which contains both close collisions and collective electromagnetic fields (see details in “Methods”). The simulation assumes, the incident proton beam to have Gaussian distribution in space and time, with a beam duration of 1 ps and a transverse extension of 1 mm. The energy spectrum is also assumed to be Gaussian, with the peak of the energy distribution at 3.36 MeV and FWHM of 0.10 MeV. The measured ionized target parameters were used as simulation input. The simulation was carried out in *Z*–*Y* Cartesian geometry with beam propagating along the *Z* direction. The size of the simulation box was 1.2 mm × 2.5 mm, with a grid size of 0.75 μm × 25 μm.

**Fig. 4:**
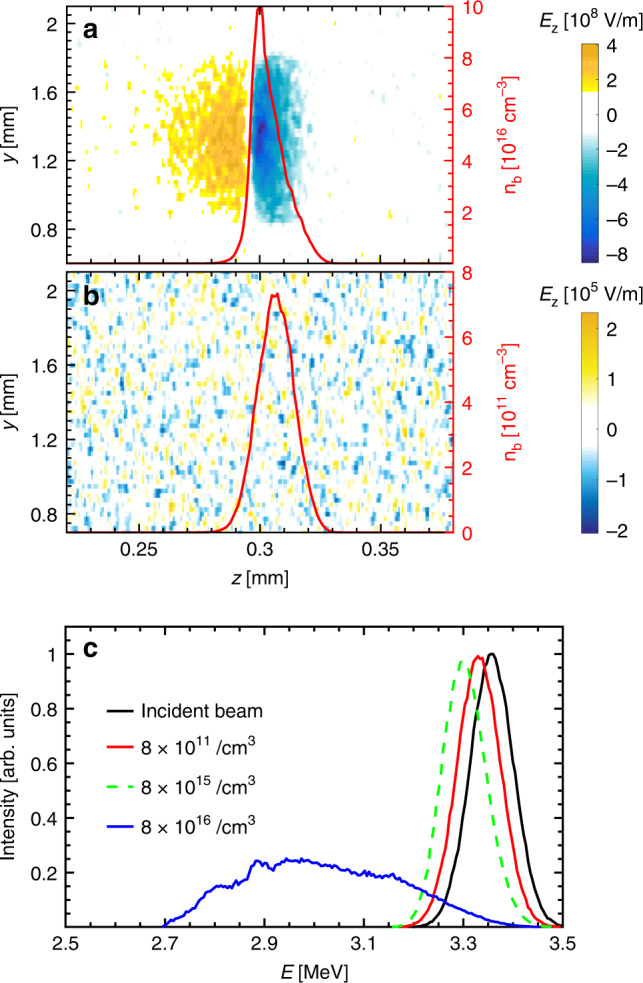
Simulated distribution of longitudinal electric field and proton beam density during beam transport in experimentally used ionized target, and the resulting proton beam energy spectra shift after passing through the sample. **a** Longitudinal electric field driven by proton beam, moving along the *Z* direction with a beam density of 8 × 10^16^ cm^−3^. The beam density profile is indicated by the red solid curve. **b** The same situation as in (**a**) but the initial beam density is reduced to 8 × 10^11^ cm^−3^. **c** Normalized energy spectra. The energy spectra of protons without target are represented by the black solid curve. The colored lines indicate the energy spectra of protons passing through the plasma target for varying beam densities of 8 × 10^11^ cm^−3^ (red solid line), 8 × 10^15^ cm^−3^ (green dashed line), and 8 × 10^16^ cm^−3^ (blue solid line).

Given the incident proton beam with density of 8 × 10^16^ cm^−3^, which corresponds to high-current case of 3 × 10^7^ A/cm^2^, Fig. 4a shows the longitudinal electric field *E*_*z*_ induced by the beam-driven return current after a propagation distance of about 0.3 mm. A strong decelerating field approaching 10^9^ V/m is generated, and contributes to the proton stopping. The proton energy spectrum after passing through 1 mm of plasma is shown in Fig. 4c. The energy spread is significantly broadened compared to the initial spread. We attribute this to a decreasing field, the protons are imbedded in. Protons with higher energies are located at the front end of bunch and therefore experience a smaller decelerating electric field than those with lower energies that come later. The spatial size of this decelerating field is comparable to the size of the proton bunch. This is different from the plasma wakefield case^81,82^, where the spatial structure of the electric field is determined by the plasma density. Here the plasma wakefield wavelength is much smaller than the beam length, therefore the wakefield-induced collective acceleration and deceleration cancel out. The central energy of proton spectrum is downshifted by 0.36 MeV after passing through the plasma. As shown in Fig. 3, this energy shift (blue triangle) agrees with experimental data in magnitude. We carried out additional simulations for different beam densities at 8 × 10^11^ cm^−3^ and 8 × 10^15^ cm^−3^, which are defined as low- and intermediate-current cases, respectively. For the low-current case, the beam-induced longitudinal electric field *E*_*z*_ after propagating for 0.3 mm in the plasma is shown in Fig. 4b. No collective decelerating field is excited under such conditions. After passing through the plasma, the energy spectrum is downshifted by only 0.03 MeV as shown in Fig. 4c. This prediction agrees well with those calculated by the different binary collision theories, as shown in Fig. 3, which indicates the dominant role of collisional stopping in low-current cases. As for the intermediate case, the stopping due to the collective effects is comparable to that caused by binary collisions, giving rise to an energy loss of 0.06 MeV as shown in Fig. 4c.

In all, the energy loss of laser-accelerated intense proton beam in dense ionized matter consists of (d*E*/d*x*)_collision_ and (d*E*/d*x*)_collective_. Bethe–Bloch, LP, SSM models, and PIC simulation for low-current case give similar predictions for (d*E*/d*x*)_collision_, which is one order of magnitude lower than the experimental data. PIC simulation for high-current cases shows that when sending a very dense ion bunch into the plasma, strong electric fields can be induced, and the ion bunch is imbedded in the deceleration field. This leads to a significant enhancement of the energy loss, which fairly well explains our observation.

In summary, the laser-accelerated intense proton beam stopping in a dense ionized matter has been measured. Benefiting from the fact that we have a quasi-monoenergetic proton beam and long-living well-characterized dense ionized target, accurate stopping power data were obtained. The measured stopping power exceeds the classical theory predictions in binary collision scheme by about one order of magnitude. The phenomenon can be very well explained by our PIC simulation combined with a Monte Carlo binary collision model and a reduced model taking account of the collective electromagnetic effects. The stopping power is dramatically enhanced due to the return-current-induced decelerating electric field approaching 1 GV/m. We have demonstrated the existence of collective effects, for high-density beam, leading to enhanced stopping. This will be important for the optimum design of ion driven inertial confinement fusion and fast ignition scenarios.

## Methods

The collisional model in the current PIC code is based on Monte Carlo binary collisions, which has the advantage of calculating the beam stopping in a natural manner. The model includes binary collisions among electron–electron, electron–ion, and ion–ion, taking into account contributions from both free and bound electrons. Compared with other existing models, physical quantities, such as angular scattering, momentum transferring, and temperature variation, can be taken into account quite readily in the approach.

In the calculations, three steps are made iteratively: (i) pair of particles are selected randomly in the cell, i.e., either electron–electron, electron–ion, or ion–ion pairs; (ii) for these pair of particles, the binary collisions are associated with changes in the velocity of the particles within the time interval *δt*, which are calculated; (iii) and then the velocity of each particle is replaced by the newly calculated one.

In order to contain both bound and free electrons’ contribution into the binary collision model, we here take the collision frequency between ions and electrons, in the above (ii) step, as,

1 $${\nu }_{\text{i}-\text{e}}=\frac{8\sqrt{2\pi }{e}^{4}{Z}_{b}^{2}Z{n}_{i}}{3{m}_{e}^{2}{\beta }^{3}}[\mathrm{ln}\,({\Lambda }_{\text{f}})+\frac{A-Z}{Z}\mathrm{ln}\,({\Lambda }_{\text{b}})],$$

where

2 $${\mathrm{ln}}\,({\Lambda }_{\mathrm{b}})\equiv {\mathrm{ln}}\,[\frac{2{\gamma }^{2}{m}_{e}{{\beta} }^{2}}{{\bar{I}}_{A}(Z)}]-{\beta }^{2}-{C}_{\text{K}}/A-\delta /2,$$

and

3 $$\mathrm{ln}\,({\Lambda }_{\text{f}})\equiv \mathrm{ln}\,({\lambda }_{\text{D}}/b).$$

*A* is the atomic number of stopping medium, *Z* is the ionization degree of background plasmas, *n*_*i*_ is the nucleus density of stopping medium, *m*_*e*_ is the electron mass, *γ* is the relativistic factor of the projected ions, *β* is the velocity of projected ions, ${\bar{I}}_{A}$ is the average ionization potential, and *Z*_*b*_ is the effective charge state of injected ion beams, which equals ‘1’ for the case of protons in our present studies. In Eq. (1), the latter two terms are the shell correction term and the density effect correction term, respectively. These two terms are based on Fano’s original work^83^, to which the definitions of *C*_K_/*A* and *δ*/2 can be referred. The Debye length, *λ*_D_, is a dynamic value changing as ${\lambda }_{\text{D}}=\sqrt{({T}_{e}/4\pi {n}_{e})(1+{\beta }^{2}/{v}_{\,\text{th}\,}^{2})}$, where *T*_*e*_ and *v*_th_ are the temperature and thermal velocity of background electrons. Parameter *b* is the distance of closest approach between the two charges. Especially, (*A* − *Z*)/*Z* defines the ratio of bound electrons’ contributions. For fully ionized plasmas, *Z* → *A*, the collision frequency between ions and electrons converges to

4 $${\nu }_{\text{i}-\text{e}} \sim \frac{8\sqrt{2\pi }{Z}_{b}^{2}{e}^{4}Z{n}_{i}}{3{m}_{e}^{2}{\beta }^{3}}\mathrm{ln}\,({\Lambda }_{\text{f}}).$$

For neutral atoms, *Z* → 0, in contrast, the frequency is

5 $${\nu }_{\text{i}-\text{e}} \sim \frac{8\sqrt{2\pi }{Z}_{b}^{2}{e}^{4}A{n}_{i}}{3{m}_{e}^{2}{\beta }^{3}}\mathrm{ln}\,({\Lambda }_{\text{b}}).$$

At the low-temperature limit, when all electrons are bound at the nucleus, the calculated stopping powers converge to the NIST ones with the average ionization degree approaching zero as the stopping powers of cold materials can be well calculated by Bethe–Bloch formula. With the increase of temperature, more and more bound electrons are ionized, giving rise to an increased stopping power to cold matter. When the temperature is further increased, with ionizations reaching the maximum, lowered stopping power is observed, which is due to the suppression of collision frequency between projected proton beam and hot plasmas in the target.

Simulation of large scale plasmas often results in an intractable burden on computer power. Therefore, instead of solving the full Maxwell’s equations, we combine the PIC method with a reduced model^79^. To take into account collective electromagnetic effects, the background electron inertia is neglected, and instead the background return current is evaluated by the Ampere’s law **J**_*e*_ = (1/2*π*) ∇ × **B** − (1/2*π*)∂**E**/∂*t* − **J**_*b*_ − **J**_*i*_, where **B** is the magnetic field, **E** is the electric field, **J**_*b*_ is the injected beam current, and **J**_*i*_ is the background ion current. Applying the continuity equation  ∇ ⋅ **J** + ∂*ρ*/∂*t* = 0 with the total current **J** = **J**_*b*_ + **J**_*i*_ + **J**_*e*_, where *ρ* is the charge density, the Poisson equation  ∇ ⋅ **E** = 2*πρ* is rigorously satisfied. The electric fields are then obtained from Ohm’s law, **E** = *η***J**_*e*_ − **v**_*e*_ × **B**, where **v**_*e*_ is the background electron velocity, and *η* is the resistivity. Taking advantage of the Monte Carlo collision model, resistivity *η* is obtained by averaging over all binary collisions at each time step for each simulation cell. Finally, Faraday’s law is used to obtain the magnetic fields ∂**B**/∂*t* = − ∇ × **E**. This field solver, which couples Ampere’s law, Faraday’s law, and Ohm’s law, can completely remove the numerical heating and reduces significantly the numerical expense. With these advantageous features a unique tool is at hand, which can self-consistently model transport and energy deposition of intense charged particles in dense ionized matter.

## Supplementary information

Supplementary Information

Peer Review File

## Data Availability

The datasets generated and analyzed during the current study are available from the corresponding authors upon reasonable request. The simulation details are available from the corresponding author on reasonable request.

## References

[1] Hurricane OA (2016). Inertially confined fusion plasmas dominated by alpha-particle self-heating. Nat. Phys..

[2] Atzeni, S. & Vehn, J. M.-T. *The Physics of Inertial Fusion: Beam Plasma Interaction, Hydrodynamics, Hot Dense Matter*, Vol. 125 (OUP, Oxford, 2004).

[3] Paradela J, García-Rubio F, Sanz J (2019). Alpha heating enhancement in MagLIF targets: a simple analytic model. Phys. Plasmas.

[4] Betti R, Hurricane OA (2016). Inertial-confinement fusion with lasers. Nat. Phys..

[5] Jacquemot S (2017). Inertial confinement fusion for energy: overview of the ongoing experimental, theoretical and numerical studies. Nucl. Fusion.

[6] Basov NG, Yu Gus’kov S, Feokistov LP (1992). Thermonuclear gain of ICF targets with direct heating of ignitor. J. Sov. Laser Res..

[7] Tabak M (1994). Ignition and high gain with ultrapowerful lasers. Phys. Plasmas.

[8] Roth M (2001). Fast ignition by intense laser-accelerated proton beams. Phys. Rev. Lett..

[9] Wang W, Gibbon P, Sheng Z, Li Y (2015). Magnetically assisted fast ignition. Phys. Rev. Lett..

[10] Fernández JC (2009). Progress and prospects of ion-driven fast ignition. Nucl. fusion.

[11] Nagatomo H (2019). Study of fast ignition target design for ignition and burning experiments. Nucl. Fusion.

[12] Ratan N (2017). Dense plasma heating by crossing relativistic electron beams. Phys. Rev. E.

[13] Yanagawa T, Sakagami H, Sunahara A, Nagatomo H (2015). Asymmetric implosion of a cone-guided target irradiated by Gekko XII laser. Laser Part. Beams.

[14] Temporal M (2011). Irradiation uniformity of directly driven inertial confinement fusion targets in the context of the shock-ignition scheme. Plasma Phys. Controlled Fusion.

[15] Hofmann I (2018). Review of accelerator driven heavy ion nuclear fusion. Matter Radiat. Extremes.

[16] Pelka A (2010). Ultrafast melting of carbon induced by intense proton beams. Phys. Rev. Lett..

[17] Hoffmann DHH (2002). Unique capabilities of an intense heavy ion beam as a tool for equation-of-state studies. Phys. Plasm..

[18] Mintsev V (2016). Non-ideal plasma and early experiments at fair: Hihex-heavy ion heating and expansion. Contrib. Plasm. Phys..

[19] Ren J (2018). Accelerator-driven high-energy-density physics: status and chances. Contrib. Plasm. Phys..

[20] Ziegler JF (1999). Stopping of energetic light ions in elemental matter. J. Appl. Phys..

[21] McGuire EJ, Peek JM, Pitchford LC (1982). Proton stopping power of aluminum ions. Phys. Rev. A.

[22] Young FC, Mosher D, Stephanakis SJ, Goldstein SA, Mehlhorn TA (1982). Measurements of enhanced stopping of 1-MeV deuterons in target-ablation plasmas. Phys. Rev. Lett..

[23] Jacoby J (1995). Stopping of heavy ions in a hydrogen plasma. Phys. Rev. Lett..

[24] Hoffmann DHH (1990). Energy loss of heavy ions in a plasma target. Phys. Rev. A.

[25] Maeder, R. et al. Measurements of the heavy ion stopping in X-ray heated low-density nanostructured targets. *GSI Annual Scientific Reports*, page 450 (2011).

[26] Li C-K, Petrasso RD (1993). Fokker-Planck equation for moderately coupled plasmas. Phys. Rev. Lett..

[27] Maynard G, Deutsch C (1985). Born random phase approximation for ion stopping in an arbitrarily degenerate electron fluid. J. Phys..

[28] Bethe H (1930). Zur theorie des durchgangs schneller korpuskularstrahlen durch materie. Ann. der Phys..

[29] Bloch F (1933). Zur bremsung rasch bewegter teilchen beim durchgang durch materie. Ann. der Phys..

[30] Brown LS, Preston DL, Singleton RL (2005). Charged particle motion in a highly ionized plasma. Phys. Rep..

[31] Gericke DO (2002). Stopping power for strong beam-plasma coupling. Laser Part. Beams.

[32] Deutsch C, Maynard G (2018). Ion stopping in dense plasmas: a basic physics approach. Matter Radiat. Extremes.

[33] Frenje JA (2019). Experimental validation of low-z ion-stopping formalisms around the Bragg peak in high-energy-density plasmas. Phys. Rev. Lett..

[34] Zylstra AB (2015). Measurement of charged-particle stopping in warm dense plasma. Phys. Rev. Lett..

[35] Cayzac W (2017). Experimental discrimination of ion stopping models near the Bragg peak in highly ionized matter. Nat. Commun..

[36] Ding YH, White AJ, Hu SX, Certik O, Collins LA (2018). Ab initio studies on the stopping power of warm dense matter with time-dependent orbital-free density functional theory. Phys. Rev. Lett..

[37] Rygg JR (2008). Proton radiography of inertial fusion implosions. Science.

[38] Gu YJ, Klimo O, Bulanov SV, Weber S (2018). Brilliant gamma-ray beam and electron-positron pair production by enhanced attosecond pulses. Commun. Phys..

[39] Vranic M, Klimo O, Korn G, Weber S (2018). Multi-GeV electron-positron beam generation from laser-electron scattering. Sci. Rep..

[40] Li Y-F (2020). Polarized ultrashort brilliant multi-GeV gamma-rays via single-shot laser-electron interaction. Phys. Rev. Lett..

[41] Kodama R (2001). Fast heating of ultrahigh-density plasma as a step towards laser fusion ignition. Nature.

[42] Cobble JA, Johnson RP, Cowan TE, Galloudec NR, Allen M (2002). High resolution laser-driven proton radiography. J. Appl. Phys..

[43] Roth M (2013). Bright laser-driven neutron source based on the relativistic transparency of solids. Phys. Rev. Lett..

[44] Patel PK (2003). Isochoric heating of solid-density matter with an ultrafast proton beam. Phys. Rev. Lett..

[45] Bartal T (2012). Focusing of short-pulse high-intensity laser-accelerated proton beams. Nat. Phys..

[46] Hegelich BM (2006). Laser acceleration of quasi-monoenergetic MeV ion beams. Nature.

[47] Cowan TE (2004). Ultralow emittance, multi-MeV proton beams from a laser virtual-cathode plasma accelerator. Phys. Rev. Lett..

[48] Roth M (2002). Energetic ions generated by laser pulses: a detailed study on target properties. Phys. Rev. Spec. Top.-Acc. Beams.

[49] Hegelich M (2002). MeV ion jets from short-pulse-laser interaction with thin foils. Phys. Rev. Lett..

[50] Hatchett SP (2000). Electron, photon, and ion beams from the relativistic interaction of petawatt laser pulses with solid targets. Phys. Plasmas.

[51] Snavely R (2000). Intense high-energy proton beams from petawatt-laser irradiation of solids. Phys. Rev. Lett..

[52] Maksimchuk A, Gu S, Flippo KA, Umstadter DP, YuBychenkov V (2000). Forward ion acceleration in thin films driven by a high-intensity laser. Phys. Rev. Lett..

[53] Sheng Z (2000). Angular distributions of fast electrons, ions, and bremsstrahlung X/gamma-rays in intense laser interaction with solid targets. Phys. Rev. Lett..

[54] Kim J (2015). Self-consistent simulation of transport and energy deposition of intense laser-accelerated proton beams in solid-density matter. Phys. Rev. Lett..

[55] Chen SN (2018). Experimental evidence for the enhanced and reduced stopping regimes for protons propagating through hot plasmas. Sci. Rep..

[56] Chou S-W (2016). Collective deceleration of laser-driven electron bunches. Phys. Rev. Lett..

[57] Honda M, Meyer-ter-Vehn J, Pukhov A (2000). Collective stopping and ion heating in relativistic-electron-beam transport for fast ignition. Phys. Rev. Lett..

[58] Tatarakis M (2003). Propagation instabilities of high-intensity laser-produced electron beams. Phys. Rev. Lett..

[59] Vauzour B (2014). Unraveling resistive versus collisional contributions to relativistic electron beam stopping power in cold-solid and in warm-dense plasmas. Phys. Plasm..

[60] Mccorkle RA, Iafrate GJ (1977). Beam-density effect on the stopping of fast charged particles in matter. Phys. Rev. Lett..

[61] Kawata S, Deutsch C, Gu YJ (2019). Peculiar behavior of Si cluster ions in a high-energy-density solid Al plasma. Phys. Rev. E.

[62] Deutsch C (1990). Interaction of ion cluster beams with cold matter and dense plasmas. Laser Part. Beams.

[63] Rule DW, Crawford OH (1984). Nature of the beam-density effect on energy loss by nonrelativistic charged-particle beams. Phys. Rev. Lett..

[64] Zhao, Y. et al. Stopping of laser-accelerated ion beam in a foam-plasma. *GSI-2018-2 REPORT: News and Reports from High Energy Density generated by Heavy Ion and Laser Beams*, page 35 (2018).

[65] Rosmej ON (2011). Heating of low-density CHO-foam layers by means of soft X-rays. Nucl. Instrum. Meth. A.

[66] Rosmej ON (2015). The hydrodynamic and radiative properties of low-density foams heated by X-rays. Plasma Phys. Contr. Fusion.

[67] Faik S (2014). Creation of a homogeneous plasma column by means of hohlraum radiation for ion-stopping measurements. High. Energy Density Phys..

[68] Mackinnon AJ (2004). Proton radiography as an electromagnetic field and density perturbation diagnostic (invited). Rev. Sci. Instrum..

[69] Borghesi M (2004). Multi-MeV proton source investigations in ultraintense laser-foil interactions. Phys. Rev. Lett..

[70] Sarri G (2012). Dynamics of self-generated, large amplitude magnetic fields following high-intensity laser matter interaction. Phys. Rev. Lett..

[71] Chung H-K, Chen MH, Morgan WL, Ralchenko Y, Lee RW (2005). FLYCHK: generalized population kinetics and spectral model for rapid spectroscopic analysis for all elements. High. Energy Density Phys..

[72] Zhang Y (2018). An angular-resolved multi-channel Thomson parabola spectrometer for laser-driven ion measurement. Rev. Sci. Instrum..

[73] Jung D (2011). Development of a high resolution and high dispersion Thomson parabola. Rev. Sci. Instrum..

[74] Rajeev R, Rishad KPM, MadhuTrivikram T, Narayanan V, Krishnamurthy M (2011). A Thomson parabola ion imaging spectrometer designed to probe relativistic intensity ionization dynamics of nanoclusters. Rev. Sci. Instrum..

[75] Peter T, Meyer-ter-Vehn J (1991). Energy loss of heavy ions in dense plasma. I. Linear and nonlinear Vlasov theory for the stopping power. Phys. Rev. A.

[76] Gericke DO, Schlanges M (2003). Energy deposition of heavy ions in the regime of strong beam-plasma correlations. Phys. Rev. E.

[77] Cayzac W (2015). Predictions for the energy loss of light ions in laser-generated plasmas at low and medium velocities. Phys. Rev. E.

[78] Chen BZ, Wu D, Ren JR, Hoffmann DHH, Zhao YT (2020). Transport of intense particle beams in large-scale plasmas. Phys. Rev. E.

[79] Wu D, Yu W, Fritzsche S, He XT (2019). High-order implicit particle-in-cell method for plasma simulations at solid densities. Phys. Rev. E.

[80] Wu D, He XT, Yu W, Fritzsche S (2018). Particle-in-cell simulations of laser-plasma interactions at solid densities and relativistic intensities: the role of atomic processes. High. Power Laser Sci. Eng..

[81] Huang C (2007). Hosing instability in the blow-out regime for plasma-wakefield acceleration. Phys. Rev. Lett..

[82] Adli E (2018). Acceleration of electrons in the plasma wakefield of a proton bunch. Nature.

[83] Fano U (1963). Penetration of protons, alpha particles, and mesons. Annu. Rev. Nucl. Sci..

