# Supplementary material for: Observation of a high degree of stopping for laser-accelerated intense proton beams in dense ionized matter
Source: Nat Commun. 2020 Oct 14;11:5157. doi: 10.1038/s41467-020-18986-5 (PMC7560615; doi:10.1038/s41467-020-18986-5)
Supplement: Supplementary file 1 — Supplementary Information [file 41467_2020_18986_MOESM1_ESM.pdf]

Supplementary Information

Jieru Ren,[1,*] Zhigang Deng,[2,*] Wei Qi,[2] Benzheng Chen,[1,3] Bubo Ma,[1] Xing Wang,[1] Shuai Yin,[1] Jianhua Feng,[1] Wei Liu,[1,4] Zhongfeng Xu,[1] Dieter H.H. Hoffmann,[1] Shaoyi Wang,[2] Quanping Fan,[2] Bo Cui,[2] Shukai He,[2] Zhurong Cao,[2] Zongqing Zhao,[2] Leifeng Cao,[2] Yuqiu Gu,[2] Shaoping Zhu,[5,2,6] Rui Cheng,[7] Xianming Zhou,[1,8] Guoqing Xiao,[7] Hongwei Zhao,[7] Yihang Zhang,[9,10] Zhe Zhang,[9,10] Yutong Li,[9,10] Dong Wu,[3,†] Weimin Zhou,[2,‡] and Yongtao Zhao[1,§]

[1]*MOE Key Laboratory for Nonequilibrium Synthesis and Modulation of Condensed Matter, School of Physics, Xi'an Jiaotong University, Xi'an 710049, China*

[2]*Science and Technology on Plasma Physics Laboratory, Laser Fusion Research Center, China Academy of Engineering Physics, Mianyang 621900, China*

[3]*Institute for Fusion Theory and Simulation, Department of Physics, Zhejiang University, Hangzhou 310058, China*

[4]*Xi'an Technological University, Xi'an 710021, China*

[5]*Institute of Applied Physics and Computational Mathematics, Beijing 100094, China*

[6]*Graduate School, China Academy of Engineering Physics, Beijing 100088, China*

[7]*Institute of Modern Physics, Chinese Academy of Sciences, Lanzhou 710049, China*

[8]*Xianyang Normal University, Xianyang 712000, China*

[9]*Beijing National Laboratory for Condensed Matter Physics, Institute of Physics, Chinese Academy of Sciences, Beijing 100190, China*

[10]*School of Physical Sciences, University of Chinese Academy of Sciences, Beijing 100049, China*

[*] These authors have contributed equally to this work.

[†] dwu.phys@zju.edu.cn

[‡] zhouwm@caep.cn

[§] zhaoyongtao@xjtu.edu.cn

I. BEAM PROPERTIES

In the experiments discussed in the main text, we have actually mounted additional Image Plates (IPs), and plastic track detectors CR39 in stack configuration along the beam trajectory to monitor the beam position and spatial distribution. The layout is schematically shown in Fig. 1 (a). An image plate with a slot is glued at the exit of the dipole. The scanned image in Fig. 1 (c) clearly shows that the laser-accelerated ions are dispersed by the magnetic field, and the slotted area has a larger size to enable the beam passing through. It needs to be noted that the IP saturates due to the high dose of laser-accelerated intense ion beams.

The selected ions have the same $m\mathbf{v}/q$ value, where m is the ion mass, $\mathbf{v}$ is the ion velocity, and q is the charge state of the ion. Therefore, different ion species have different velocities, so that different ion species will arrive at the target with different time delays, as is shown in Table I, which is in reference to protons. It can be seen that the carbon ions lag behind the protons by tens to hundreds of nanoseconds.

TABLE I. The velocities and relative time delays of different ion species arriving at the target.

Ion species	H^{1+}	C^{4+}	C^{3+}	C^{2+}	C^{1+}
Velocities(cm/ns)	2.5	0.8	0.6	0.4	0.2
Time delays in relative to H^{1+} (ns)	0	29.8	44.3	73.5	161.0

After passing through the dipole, series of mono-energetic ion bunches with transverse size of 0.5 mm are generated. Two CR39 detectors are mounted on the beam trajectory to record beam divergence. After a flying distance of 17 cm from dipole to CR39 I and 33 cm to CR39 II, the beam transversely spreads to 2.0 mm (Fig. 1 (d)) and 3.4 mm (Fig. 1 (e)) respectively. This consistently corresponds to a half-divergence angle of 4.4 mrad, which is orders of magnitude smaller than the previously-reported divergence angle of laser-driven ion source because the dipole acts on the charged particles.

Due to the high current of the beam, the etched tracks on the surface of the CR39 I and CR39 II show saturation. Here we only make a quite rough estimation of the beam density. Previous target normal sheath acceleration (TNSA) experiments carried out at XGIII with the same laser-target scheme show that the number of 3 MeV protons reach magnitude of 10^{13-14} per MeV. This equals to an initial density in magnitude of 10^{19-20}

/cm^3 in the selected energy range, given all the ions were generated from a diameter of 50 μm at the rear-side of the target with a duration of 1 ps. In the current experiment, the target is exactly mounted at the position of CR39 I. The divergence measurement shows that the beam current is reduced by one order of magnitude from the dipole to the target. Assuming the beam current reduction by two orders of magnitude from the source to the dipole exit and longitudinal stretching by another two orders of magnitude from source to target, the beam density is reduced by fives orders of magnitude totally from source to target. Therefore, we roughly estimate the beam density at the target to be 10^{14-15} /cm^3. The Particle In Cell simulation shows that collective stopping dominates at higher density. More experimental data and theoretical efforts investigating the dependence of energy loss on beam density are necessary to establish the discrepancy.

CR39 III coupled to TPS is used to detect the beam energy. The etched tracks (dots) on CR39 III are shown in Fig. 1 (f) together with theoretical deflection lines of different ions, which has been discussed in the main text. The energy resolution of the TPS at a specific energy E is usually characterized by E/δE, where δE is the energy range covered by the incident beam spot on the detector. In the non-relativistic case, E/δE $\approx y/2s$, where y is the deflection distance proportional to the square root of the mean energy and s is the beam spot size on the detector depending on the pinhole size and the distances from the ion source to the pinhole and the detector plane [1, 2]. As shown in Fig. 2, the selected mono-energetic proton beam has the spot size (FWHM) of 140 μm. This corresponds to δE of 0.10 MeV and the energy resolution of E/δE $\sim$ 34 at 3.36 MeV. What's more, the energy resolution is calculated via the equation E/δE $\approx y/2s$, where y is measured to be 9.5 mm for 3.36 MeV protons, and s is measured to be 140 μm in magnetic deflection direction. In this way, the same resolution of E/δE $\sim$ 34 is obtained.

II. TARGET CHRACTERIZATION

A. characterization of foam density

The foam target is fabricated with in-situ consolidation technology by the staff of Laser Fusion Research Center. Through measuring the mass and size of reference samples with regular shape, the density is determined to be 2.0 $\pm$ 0.1 mg/cm^3. The density is validated

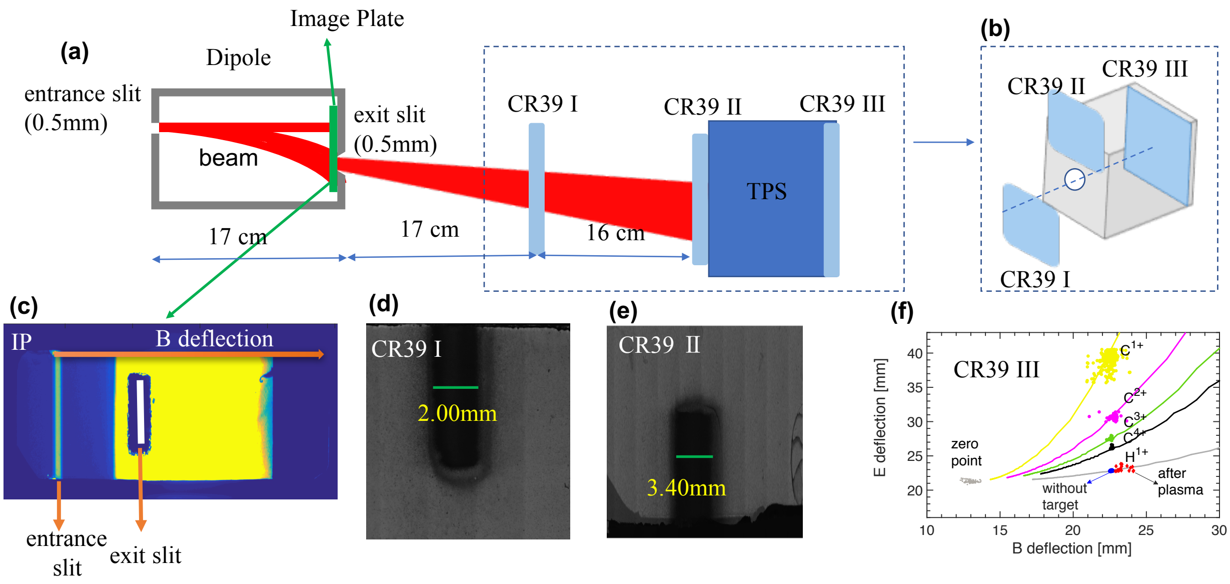

FIG. 1. (a) Top-view of the experimental setup for the beam characterization. Compared with the experimental setup in Fig. 1 of the main text, CR39 I is mounted exactly at target position to monitor the beam properties at target, and CR39 II is glued next to the entrance hole of TPS to monitor the beam properties entering TPS. (b) side view of the dashed part in (a); (c) scanned image of IP plate at the exit of the dipole. (d) etched tracks on CR39 I; (e) etched tracks on CR39 II; (f) etched tracks on CR39 III, which has been discussed in the main text.

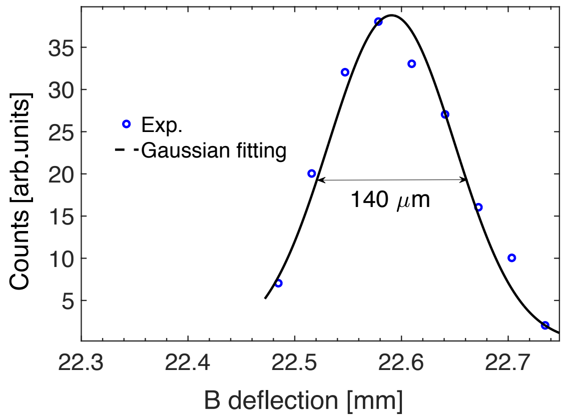

FIG. 2. The spatial distribution of tracks on CR39 III in magnetic deflection direction for protons without target.

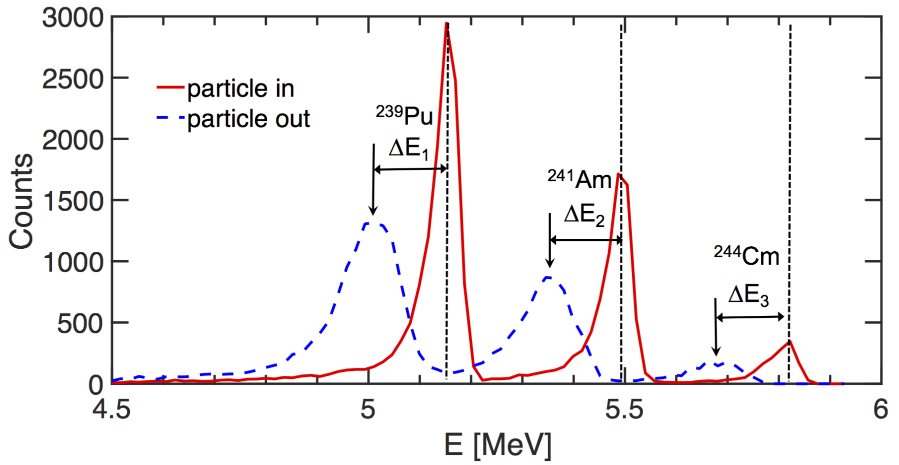

FIG. 3. Measured spectra of alpha particles from radiative sources (red solid line) and the spectra after passing through the sample (blue dashed line). The central energies of alpha particles from ^{239}Pu, ^{241}Am, ^{244}Cm are downshifted by about Δ E_1 = 0.16 MeV, Δ E_2 = 0.15 MeV, and Δ E_3 = 0.14 MeV respectively after passing through the sample.

through analysing the stopping power of the foam interacting with 5.16 MeV, 5.49 MeV, and 5.80 MeV alpha particles from ^{239}Pu, ^{241}Am, and ^{244}Cm, respectively. The energy spectra downshift of the alpha particles passing through the 1mm foam sample are shown in Fig. 3. The energy losses are determined as the central energy downshift of the spectra. Through comparing the measured energy losses with the SRIM predictions, the foam density is determined as 1.9 ± 0.3 mg/cm^3.

B. characterization of plasma temperature

Radiation spectra of hohlraum and CHO plasma are measured for the temperature characterization. A transmission grating spectrometer (TGS) with 1000 lines/mm grating, is coupled to a charge coupled device (CCD) for hohlraum radiation measurement. It needs to be mentioned that the TGS is fitted with a single-order diffraction grating, which has the advantage of filtering high-order components. The raw data, recorded by an image plate of the TGS is shown in subfigure of Fig. 4 (a). The zero order of diffraction is in the center. The first order of diffraction is analysed to reconstruct the spectrum, which is presented in Fig.4(b). The spectral resolution is $\delta\lambda$=0.8 nm at 12 nm. The emission spectrum can be well approximated by Planckian distribution with maximum intensity at λ_{max} =12.5 nm.

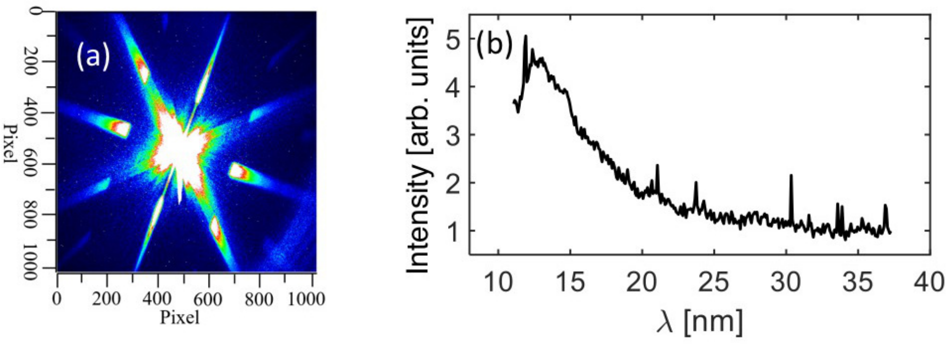

FIG. 4. (a) The raw image recorded by the image plate of TGS.(b) The measured radiation spectra of hohlraum.

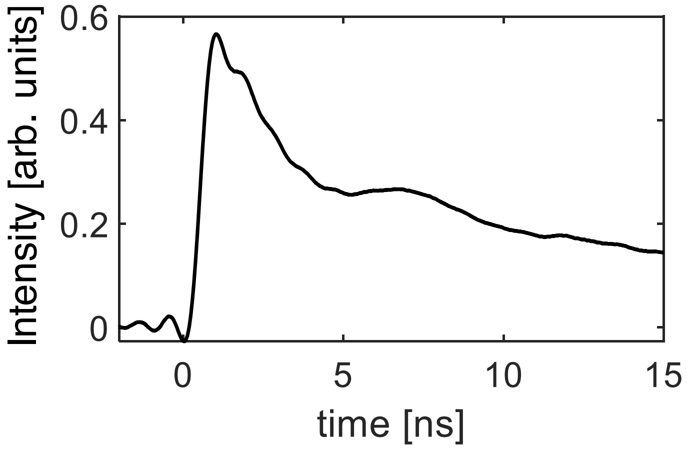

FIG. 5. Hohlraum radiation (<400 eV) flux measured by X-ray diode.

This corresponds to a black temperature of 20 eV.

To ensure the plasma is fully heated during the energy loss measurement, temporal evolution of the soft X-ray flux (<400 eV) generated by the hohlraum is measured using an X-ray diode (XRD) with carbon cathodes covered by a filter. As shown in Fig. 5, the duration of the hohlraum soft X-ray radiation is about 6 ns, during which the foam is continually heated. Therefore, in the experimental measurement, the proton beam is synchronized to arrive at the target with 10-ns delay after triggering the heating process.

In order to diagnose the CHO plasma temperature, a flat-field grating spectrometer with 1200 lines/mm is used to record the emission spectra. With a collimation slit width of 0.2 mm, the energy resolution is $\delta\lambda$=0.06 nm at 15 nm. Spectrum in the range of 13-23 nm

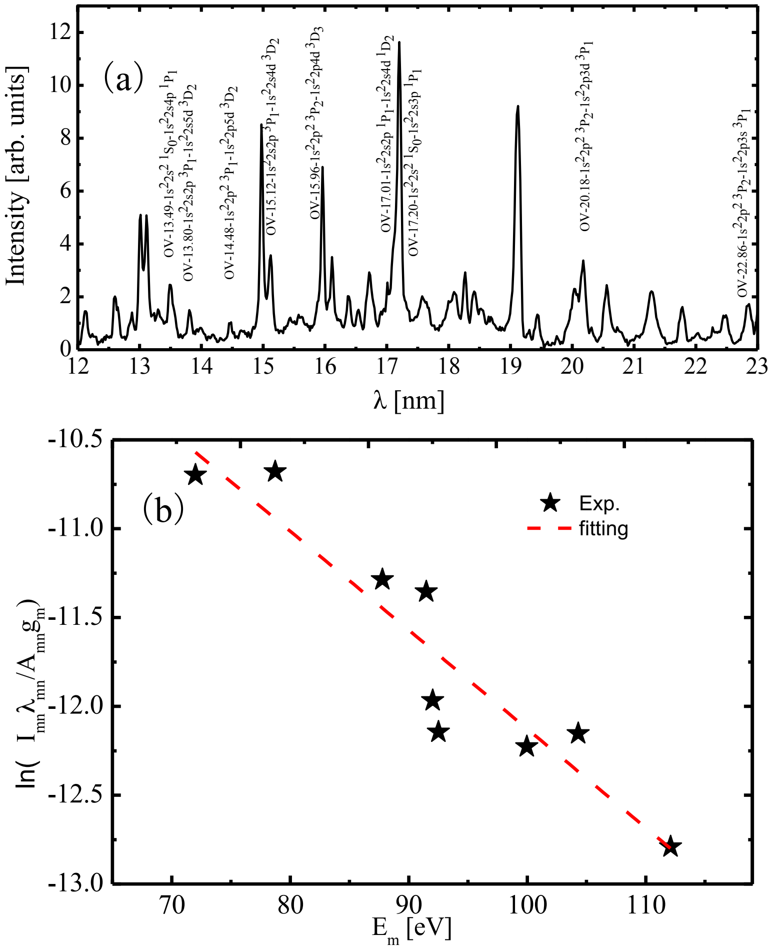

FIG. 6. (a) Emission lines of CHO plasma in range of 12-23 nm. (b) Saha-Boltzmann plotting of the emission lines. The experimental data (symbols) are linearly fitted (dashed line).

recorded with image plate is shown in Fig.6 (a). When the plasma is in local thermodynamic equilibration, the intensities of emission lines meet $ln(\frac{I_{mn}\lambda_{mn}}{A_{mn}g_m}) = -\frac{E_m}{kT_e} + C$, where I_{mn} and λ_{mn} are the photon intensity and wavelength emitted when the electron transits from upper shell m to lower shell n, E_m is the binding energy of electron in upper level, T_e is the electron temperature, C is constant, b is Boltzmann constant, A_{mn} is the transitional probability, and g_m is the statistic weight. Through performing linear fitting of $ln(\frac{I_{mn}\lambda_{mn}}{A_{mn}g_m})$ versus E_m for oxygen lines, the electron temperature is determined to be around 17 eV.

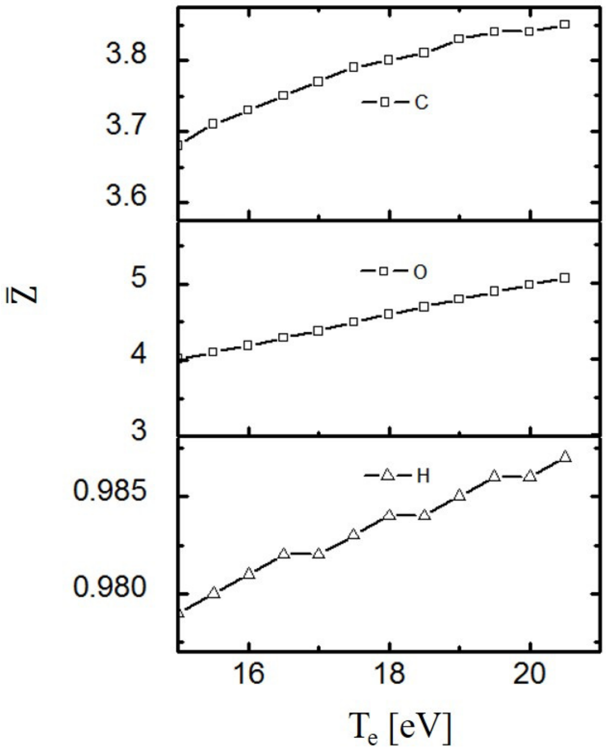

FIG. 7. Average ionization degree of the C, H and O elements in the plasma versus electron temperature.

C. characterization of plasma electron density

As investigated previously, the foam target approximately keeps its initial mass density for more than 10 ns after heating. Under such an assumption, the ionization degree of the foam is calculated by FLYCHK code. As shown in Fig. 7, the average ionization state of the CHO plasam is around $C^{3.8+}H^{0.98+}O^{4.5+}$ at electron temperature of 17 eV. This corresponds to an electron density of 4×10^{20} cm^{-3}.

III. MEASUREMENT OF INTENSE PROTON BEAM STOPPING IN TARGET

A. stopping of intense broad-band proton beams

In addition to the measurements that were discussed in the main text, another experimental campaign was carried out measuring the stopping of laser-accelerated broad-band proton beam. The measurement was carried out at XGIII as well. The schematic of the

experimental setup is shown in Fig. 8. The laser intensity, pulse duration and the plasmas were the same with that discussed in the main text. Intense heavy ions are generated following interaction of ps laser with CH-coated Cu foil through TNSA mechanism. The energy of the ions, passing through the foam target, are measured with a multi-channel TPS in conjunction with IP detector. An empty channel is drilled to allow the ions, which serves as reference ions, passing through without any disturbance simultaneously. These reference ions are supposed to have the same energy as those entering the foam, because the positions for the empty channel and foam were symmetric in relative to beam incidence.

The parabola spectra of protons passing through the system are shown in Fig. 9 (a). The deflected distances are converted to energies in Fig. 9 (b). The measured energy spectra of protons passing through the CHO plasma (blue solid curves) were obviously downshifted compared with that passing through the empty channel (cyran solid curve). The energy downshift is in principle the energy loss of protons in the plasma. The energy spectra of protons passing through the plasma are calculated by Bethe-bloch formula (black dashed curve). In the calculation, the incident protons has the same spectra as that measured after passing through the empty channel. It can be found that the measured energy downshift, namely the energy loss, is greatly enhanced compared with Bethe-bloch predictions.

It needs to be noted that the energy spectra downshift is much more significant than that measured with mono-energetic proton beam. This is attributed to the fact that here the beam intensity is much higher because of the closer distance between the source and target, and the collective stopping effect, which is nearly proportional to the beam density is expected to be more prominent. Unfortunately, since the laser-accelerated ions had typically broad bandwidth, it is hard to interpret the result quantitatively. Therefore, using the dipole to generate mono-energetic proton beam was highly desirable for precise energy loss measurement.

B. stopping of intense mono-energetic proton beams

The experimental layout for intense mono-energetic proton beam stopping measurement is shown in Fig. 10 (a). Plastic track detectors CR39 I and CR39 II are pasted below the foam target and around the entrance hole of TPS respectively to characterize the beam before and after passing through target. The results in Fig. 10 (c), (f) and (i) correspond

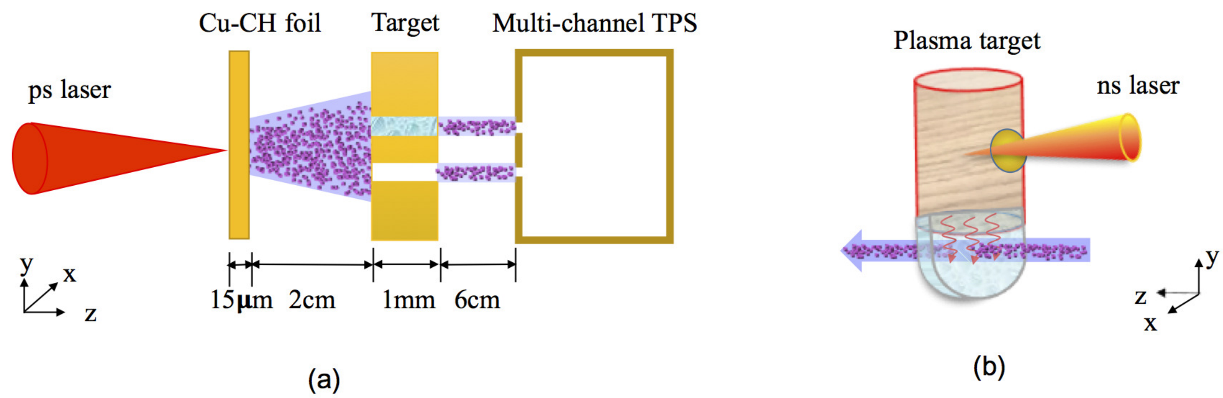

FIG. 8. (a) Experimental scenario for the measurement of laser-accelerated ions in dense plasma. (b)Target scheme for generating dense plasma.

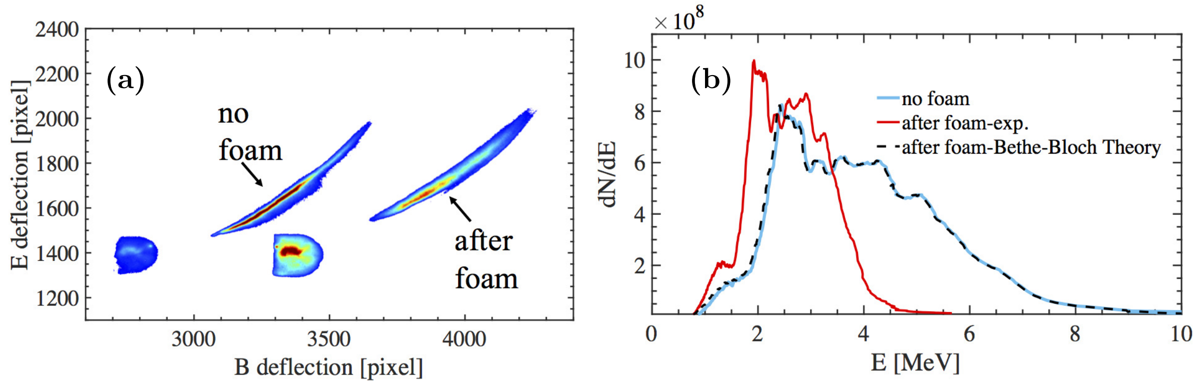

FIG. 9. (a) The scanned IP detector image of protons passing through the system shown in Fig.8. The left curve indicated the protons passing through the blank hole, and the right indicated protons passing through the plasma. (b) Measured energy spectra of protons passing through the blank hole (cyran solid curve) and plasma (red solid curve). For comparison, Bethe-bloch predictions of protons passing through plasma is shown as well by black dashed curve. In the theoretical calculations, the input beam was supposed to have the same energy spectra with that measured in case of protons passing through the blank hole.

to the shot measuring stopping power of proton beam in cold foam target. The results in Fig. 10 (d), (g) and (j) correspond to the shot measuring stopping power of proton beam in plasma. For comparison, the results without any target, which has been discussed in the beam property section, are shown again in Fig. 10, (b), (e) and (h).

As shown in Fig. 10 (b), (c) and (d) , the CR39 I detectors saturate and have similar

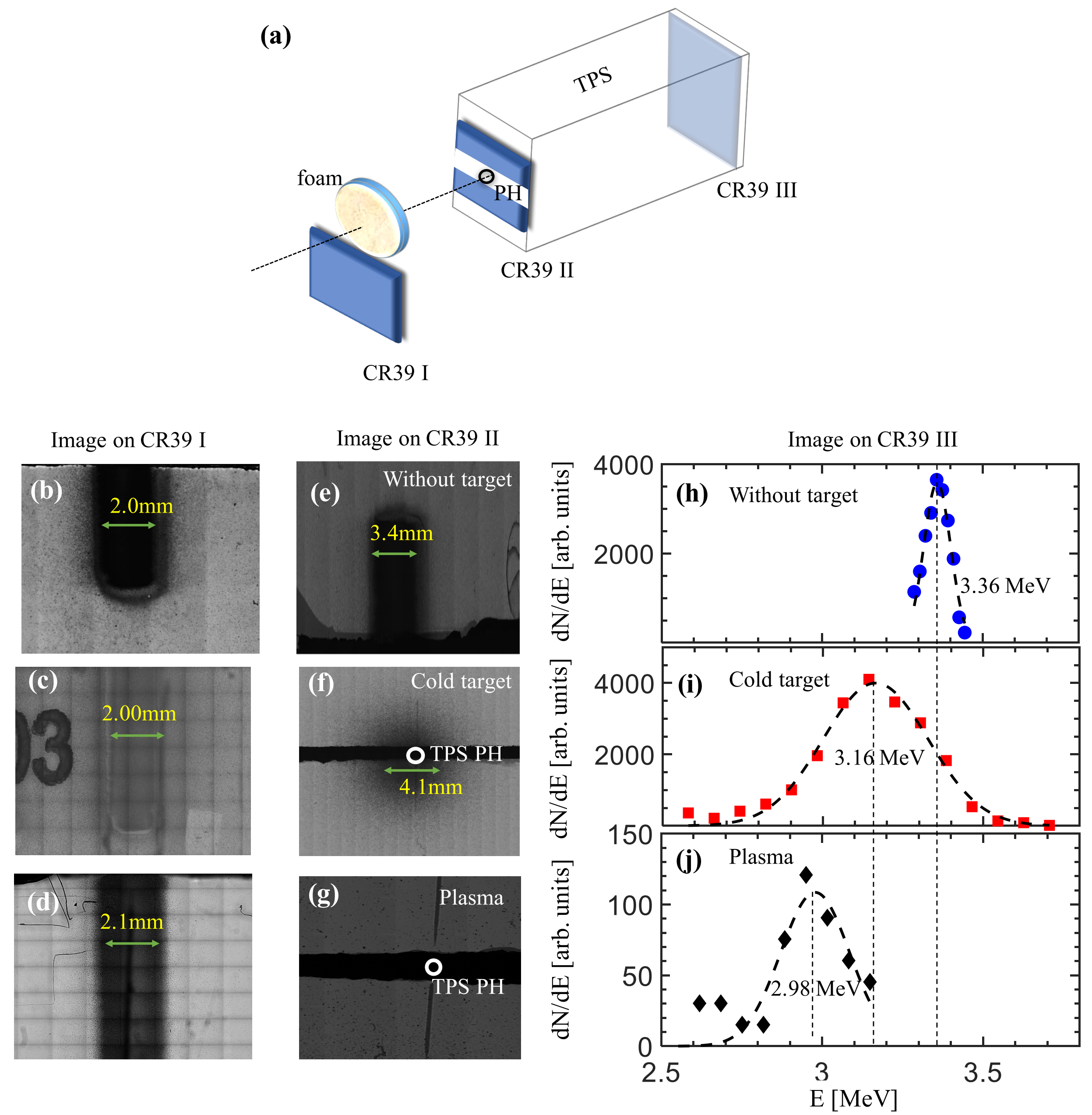

FIG. 10. (a) Experimental layout. Compared with Fig. 1 in the main text, additional CR39 I and CR39 II are mounted in front of foam target and TPS respectively to measure the beam position and distribution; (b-d) Etched tracks on CR39 I in cases of null target, cold target and plasma target respectively; (e-g) Etched tracks on CR39 II in cases of null target, cold target and plasma target respectively. The white circles in (f) and (g) represent the position of TPS pinhole; (h-j) Proton energy spectra in cases of null target, cold target and plasma target respectively.

spatial size, indicating high beam current and similar divergence for the three shots. After passing through cold target, the beam size spreads from the foam size (1 mm) to 4.08 mm at CR39 II. This corresponds to a half-divergence angle of 9.6 mrad, which is 2 folds larger than the incident beam due to atomic collisions in the target. The beam energy spectra are downshift by 0.2 MeV, as shown in Fig. 10 (h) and (i). Comparatively, after passing through plasma, the number of tracks is greatly reduced, as shown in Fig. 10 (g). This indicates the beam divergence gets much larger.

[1] D. Jung, R. Hrlein, D. Kiefer, S. Letzring, D. C. Gautier, U. Schramm, C. Hbsch, R. hm, B. J. Albright, J. C. Fernandez, D. Habs, and B. M. Hegelich. Development of a high resolution and high dispersion thomson parabola. *Review of Scientific Instruments* 82, 013306 (2011).

[2] R. Rajeev, K. P. M. Rishad, T. Madhu Trivikram, V. Narayanan, and M. Krishnamurthy. A thomson parabola ion imaging spectrometer designed to probe relativistic intensity ionization dynamics of nanoclusters. *Review of Scientific Instruments* 82, 083303 (2011).
